# Design and rationale for the prospective treatment efficacy in IPF using genotype for NAC selection (PRECISIONS) clinical trial

**DOI:** 10.1186/s12890-022-02281-8

**Published:** 2022-12-13

**Authors:** Anna J. Podolanczuk, John S. Kim, Christopher B. Cooper, Joseph A. Lasky, Susan Murray, Justin M. Oldham, Ganesh Raghu, Kevin R. Flaherty, Cathie Spino, Imre Noth, Fernando J. Martinez, for the PRECISIONS Study Team, Elizabeth Freiheit, Adam Martin-Schwarze, Ashley Szparza, Tanvi Naik, Rex Edwards, Gordon Bernard, Deborah Barnbaum, Joao de Andrade, Daren Knoell, Peter Lindenauer, Andre Rogatko, Marinella Temprosa, Shwu-Fan Ma, Emma Strickland, Jamie Sheth, Joyce Lee, Cheryl Nickerson-Nutter, David Lebo, Elizabeth Belloli, Candace Flaherty, Timothy Whelan, Max Lento, Amy Case, Ugonna Nwosu, Matthew Kottmann, Gerard Criner, Julie Juhas, Joshua Mooney, Jeanette Smith, Andrew Limper, Shannon Daley, Tessy Paul, Yousef Althulth, Chad Newton, Rhoda Annoh Gordon, Mary Strek, Spring Maleckar, Hyun Kim, Mandi DeGrote, Reba Blissell, Robert Kaner, Elizabeth Peters, Alicia Morris, Mark Hamblin, Carime Ward, Ryan Boente, Meghan Willig, Nitin Bhatt, Benjamin Hood, Cathleen Wilson, Sachin Chaudhary, Heidi Erickson, Haylie Lengel, Daniel Dilling, Sydney Montesi, Caroline Fromson, Toby Maher, Anoop Nambiar, Hilda Pomroy, Mary Beth Scholand, Chloe Kirkpatrick, Lisa Lancaster, Jim Del Greco, Stephen Sam Weigt, Eileen Callahan

**Affiliations:** 1 Department of Medicine, Weill Cornell Medical College, 1305 York Ave, Box 96, New York, NY 10021 USA; 2 Department of Medicine, University of Virginia School of Medicine, Charlottesville, VA USA; 3 Department of Medicine and Department of Physiology, University of California Los Angeles David Geffen School of Medicine, Los Angeles, CA USA; 4 Deparment of Medicine, Tulane University School of Medicine, New Orleans, LA USA; 5 Department of Biostatistics, University of Michigan, Ann Arbor, MI USA; 6 Deparment of Medicine, University of Michigan, Ann Arbor, MI USA; 7 Department of Medicine and Department of Laboratory Medicine and Pathology, University of Washington, Seattle, WA USA

**Keywords:** IPF, Clinical trial, Protocol, N-acetylcysteine

## Abstract

**Background:**

Idiopathic pulmonary fibrosis (IPF) is a progressive lung disease with few treatment options. *N*-acetylcysteine (NAC) is a well-tolerated, inexpensive treatment with antioxidant and anti-fibrotic properties. The National Heart, Lung, and Blood Institute (NHLBI)-sponsored PANTHER (Prednisone Azathioprine and NAC therapy in IPF) trial confirmed the harmful effects of immunosuppression in IPF, and did not show a benefit to treatment with NAC. However, a post hoc analysis revealed a potential beneficial effect of NAC in a subgroup of individuals carrying a specific genetic variant, *TOLLIP* rs3750920 TT genotype, present in about 25% of patients with IPF. Here, we present the design and rationale for the Phase III, multi-center, randomized, double-blind, placebo-controlled Prospective Treatment Efficacy in IPF Using Genotype for NAC Selection (PRECISIONS) clinical trial.

**Methods:**

The PRECISIONS trial will randomize 200 patients with IPF and the *TOLLIP* rs3750920 TT genotype 1:1 to oral *N*-acetylcysteine (600 mg tablets taken three times a day) or placebo for a 24-month duration. The primary endpoint is the composite of time to 10% relative decline in forced vital capacity (FVC), first respiratory hospitalization, lung transplantation, or death from any cause. Secondary endpoints include change in patient-reported outcome scores and proportion of participants with treatment-emergent adverse events. Biospecimens, including blood, buccal, and fecal will be collected longitudinally for future research purposes. Study participants will be offered enrollment in a home spirometry substudy, which explores time to 10% relative FVC decline measured at home, and its comparison with study visit FVC.

**Discussion:**

The sentinel observation of a potential pharmacogenetic interaction between NAC and *TOLLIP* polymorphism highlights the urgent, unmet need for better, molecularly focused, and precise therapeutic strategies in IPF. The PRECISIONS clinical trial is the first study to use molecularly-focused techniques to identify patients with IPF most likely to benefit from treatment. PRECISIONS has the potential to shift the paradigm in how trials in this condition are designed and executed, and is the first step toward personalized medicine for patients with IPF.

*Trial Registration* ClinicalTrials.gov identifier: NCT04300920. Registered March 9, 2020. https://clinicaltrials.gov/ct2/show/NCT04300920

**Supplementary Information:**

The online version contains supplementary material available at 10.1186/s12890-022-02281-8.

## Background

Idiopathic pulmonary fibrosis (IPF) is a chronic respiratory disease characterized by progressive scarring and architectural distortion of the lung parenchyma [1]. Patients with IPF have high morbidity, poor quality of life, and an average survival of 3–5 years from diagnosis [2]. There are only two Food and Drug Administration (FDA)-approved treatments for IPF in the United States, pirfenidone and nintedanib. Both drugs slow disease progression and have been linked to reduced mortality [3–5]. However, neither pharmacologic therapy reverses fibrosis or improves patient symptoms, and both drugs carry a significant side effect profile, making them poorly tolerated by a significant proportion of patients. Therefore, there is a continued, urgent, unmet need for more effective and better tolerated therapies for IPF.

In this protocol paper, we describe the design and rationale of the Phase III, multi-center randomized, double-blind, placebo-controlled Prospective Treatment Efficacy in IPF Using Genotype for NAC Selection (PRECISIONS) clinical trial. We highlight the novel approach of this trial to patient selection, by identifying individuals that carry a specific genetic variant, *TOLLIP* rs3750920 TT genotype, who are most likely to benefit from *N*-acetylcysteine (NAC) therapy. The PRECISIONS trial is one of the first studies to use a precision medicine approach in IPF, by testing the efficacy of a therapy in a targeted population based on a specific genotype.

## Methods

### Rationale

NAC is an inexpensive and well-tolerated agent for treatment of lung and other organ diseases [6]. There has been long-standing interest in the potential therapeutic properties of NAC in IPF. NAC increases production of the antioxidant glutathione and has demonstrated anti-fibrotic properties [7]. Oral administration of NAC in IPF patients leads to higher levels of glutathione in bronchoalveolar lavage fluid, and reduced markers of oxidative stress at the alveolar surface [8, 9]. The IFIGENIA trial examined the efficacy of NAC added to standard of care, which at the time included treatment with prednisone plus azathioprine, compared to standard of care alone, on disease progression in IPF. Therapy with NAC at 600 mg three times daily added to prednisone and azathioprine slowed the decline in FVC and single-breath carbon monoxide diffusing capacity (DLCO) at one year [10].

The IPFnet PANTHER trial compared the effect of NAC alone or NAC with prednisone and azathioprine versus placebo on lung function decline in IPF [11]. There was increased mortality observed in the combination therapy arm and this arm was terminated early. There was no difference in outcomes between the NAC-only arm and placebo in the overall study population [12]. However, a post hoc analysis suggested that shifting patterns in participant enrollment and characteristics over the course of the study, and specifically after the early termination of the immunosuppressive therapy arm, may have influenced the observed results. This leaves open the possibility that a subgroup of patients with IPF may benefit from NAC.

This rationale led to the candidate genotyping of polymorphisms in *TOLLIP* and *MUC5B* in the PANTHER trial cohort. A significant interaction between the *TOLLIP* rs3750920 genotype and NAC therapy was found [13]. Participants that carried the *TOLLIP* TT genotype (about 25% of the cohort) and received NAC had a significant reduction in the composite endpoint of death, transplant, hospitalization or ≥ 10% FVC decline compared with those who received placebo. In contrast, participants with the *TOLLIP* CC genotype (about 25% of the cohort) showed a trend toward harm from NAC treatment. Those with a CT genotype (about 50% of the cohort) had similar outcomes to those treated with placebo. These findings were validated in an additional independent cohort [13].

The *TOLLIP* gene encodes an ubiquitin-binding protein that regulates innate immune response by inhibiting Toll-like receptor (TLR) signaling [14]. Variants in *TOLLIP* have been linked with IPF susceptibility and mortality in patients of European ancestry [15]. TLRs play a critical role in the innate immune response to various pathogen-associated molecular patterns (PAMPs). Alterations in TLR expression and signaling have been linked to IPF disease progression and mortality [16–18]. The *TOLLIP* rs3750920 TT genotype results in increased expression of *TOLLIP* in human monocytes, which may influence TLR signaling and host response to immunomodulatory therapies [19]. These findings suggest a potential mechanism for the heterogeneity of treatment effect based on the *TOLLIP* genotype observed in PANTHER, and that NAC therapy may provide clinical benefit in a significant number of patients with IPF.

### Study overview

**Fig. 1:**
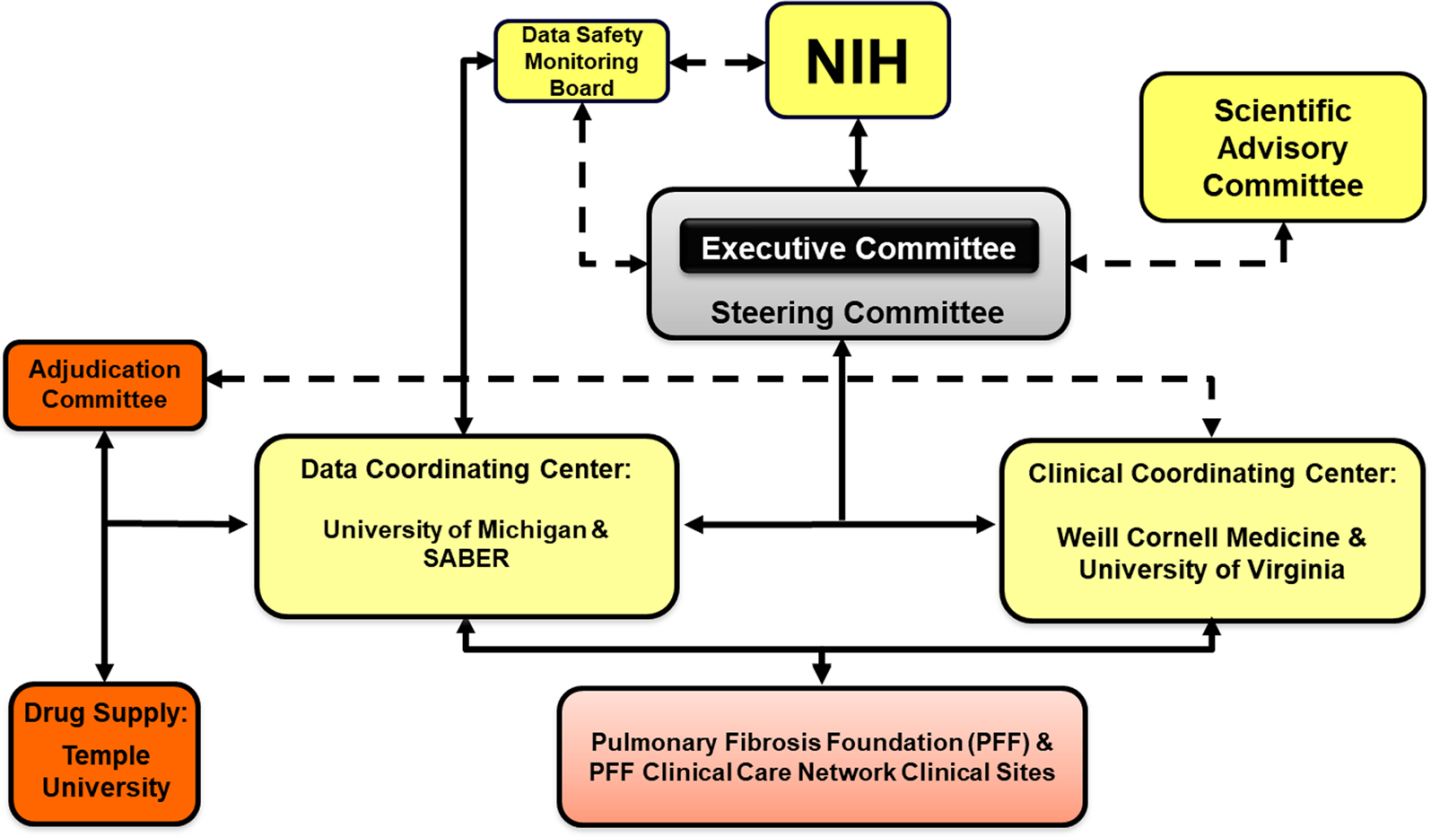
Schematic overview of the organizational structure for the PRECISIONS clinical trial. *SABER* Statistical Analysis of Biomedical and Educational Research unit, *NIH* National Institute of Health

PRECISIONS is a multi-center, randomized, double-blind, placebo-controlled trial of 200 participants with TOLLIP rs3750920 TT genotype to receive NAC or placebo for a 24-month duration. The structure of the study is shown in Fig. 1. Study participants will be randomized to NAC or placebo in a 1:1 fashion, while receiving standard of care. Standard of care is defined as allowing background therapy with FDA-approved medications for IPF, such as pirfenidone or nintedanib, if taking a stable dose for at least 6 weeks prior to enrollment. Randomization will be stratified by stable concomitant IPF therapy use (pirfenidone or nintedanib) versus no pirfenidone or nintedanib use. A schematic overview of the study design is shown in Fig. 1.

### Hypothesis

Patients with idiopathic pulmonary fibrosis (IPF) that have the *TOLLIP* rs3750920 TT genotype will exhibit improved clinical outcomes when treated with *N*-acetyl cysteine (NAC) compared to placebo, while receiving standard care.

### Study objective

The primary objective is to compare the effect of NAC plus standard care versus matched placebo plus standard care on the time to a composite endpoint of relative decline in lung function [10% relative decline in FVC, first respiratory hospitalization, lung transplantation, or all-cause mortality] in patients diagnosed with IPF who have the *TOLLIP* rs3750920 TT genotype. The secondary objectives will be to examine the effect of NAC on the components of the primary composite endpoint, the rates of clinical events, change in physiology, change in health status, and change in respiratory symptoms.

### Eligibility

The inclusion and exclusion criteria are listed in Table 1. Eligible participants must have a clinical diagnosis of IPF and the *TOLLIP* rs3750920 TT genotype. The exclusion criteria are primarily related to contraindications to receiving NAC. Participants will be recruited from approximately 25 sites in the US selected primarily from the Pulmonary Fibrosis Foundation (PFF) Care Center Network.

**Table 1:** Inclusion and Exclusion Criteria

*Inclusion Criteria*
40 years of age or greater
Diagnosed with IPF according to 2018 ATS/ERS/JRS/ALAT confirmed by the enrolling investigator
Signed informed consent
If taking pirfenidone or nintedanib, must be on stable dose for at least 6 weeks prior to the enrollment visit
Confirmed TOLLIP rs3570920 TT genotype
*Exclusion Criteria*
Pregnancy or planning to become pregnant
Significant medical, surgical or psychiatric illness that in the opinion of the investigator would affect subject safety, including liver and renal failure
Receipt of an investigational drug or biological agent within the previous 4 weeks of the screening visit or 5 times the half-life, if longer
Supplemental or prescribed NAC therapy, within 60 days of enrollment
Listed for lung transplantation at the time of screening
History of lung cancer
Inability to perform spirometry
Forced vital capacity < 45% predicted using the global lung function index equation at Visit 1
Active respiratory infection requiring treatment with antibiotics within 4 weeks of Visit 1

### Determination of TOLLIP genotype

A portion of participants we plan to enroll in PRECISIONS will have confirmed *TOLLIP* rs3750920 TT genotype through their participation in the PFF Registry and Biorepository. Samples from PFF Registry participants who previously consented to DNA genotyping and to being directly contacted for future enrollment in clinical trials will be sent coded to the University of Virginia for *TOLLIP* genotyping. The Clinical Coordinating Center (CCC) will notify study sites of potential participants with confirmed TT genotype for potential enrollment into the PRECISIONS trial. Additionally, to reach a recruitment target of 200 randomized participants, we will recruit participants with IPF and unknown *TOLLIP* genotype. These potential participants will undergo a screening visit, in which a blood sample will be collected for genotyping after informed consent is obtained. The University of Virginia School of Medicine, with supervision from Dr. Imre Noth’s laboratory, will determine the *TOLLIP* rs3750920 genotype for all blood samples collected from the genotype screening visits, and will report back to sites within 14 days.

### Study intervention

Participants will be randomized to NAC (600 mg tablets to be taken three times a day by mouth) or placebo for 24 months. Zambon Pharma (Vicenza, Italy) will supply the study drug and matching placebo (white, round tablets in blister packaging). The study drug distribution to enrolling institutions will be overseen by the investigational drug pharmacy at Temple University. Participants will initially receive a 4-month supply upon their randomization visit and 4-month refills at subsequent 4-month study visits.

### Safety review

The most frequent adverse events related to the oral use of NAC are gastrointestinal (nausea, vomiting, diarrhea, abdominal pain, nausea, stomatitis) [20]. NAC may have a detrimental effect on the stomach’s mucosa which may be relevant to patients with active peptic ulcers. Less frequent events that have been reported include anaphylactic shock, anaphylactoid reaction, bronchospasm, angioedema, dyspnea, tachycardia, rash, pruititis, and urticaria [21]. Headache, tinnitus, pyrexia, hypotension, facial edema and hemorrhage have also been reported with oral NAC. NAC has been shown to reduce platelet aggregation, although the clinical significance of this is unknown [22].

The PANORAMA study showed that IPF patients receiving both oral NAC and pirfenidone experienced higher rates of photosensitivity rash compared to those that received pirfenidone only (9% vs. 2%). However, these rates were markedly lower than the rates reported in pooled phase III trial data (33%), suggesting that this difference in the PANORAMA study may have been driven by its smaller sample size [23]. PANORAMA and a small study from Japan that examined the efficacy and tolerability of combination therapy with inhaled NAC and pirfenidone versus pirfenidone alone had findings that suggested more rapid disease progression in the group that received combination therapy [24]. The *TOLLIP* TT genotype of participants in both studies was unknown and has been shown to have a very low frequency among Japanese IPF patients. In addition, differences in dosage, modes of delivery for NAC and smaller sample sizes make the collective interpretation of these studies’ findings difficult [25].

### Procedures

At the enrollment study visit, the study coordinator will obtain and provide the following information:

- Informed consent
- Demographics
- Eligibility Review
- Medical History
- Complete Physical Exam
- Vital Signs
- Study Site Spirometry
- Home Spirometry Tutorial
- DLCO
- Quality-of-life questionnaires (Leicester Cough Questionnaire [26], EuroQoL EQ-5D [27], UCSD-SOB [28], K-BILD [29, 30], SGRQ scores [31, 32], R-Scale-PF [33])
- COVID-19 Questionnaire (Additional file 1)
- Blood collection (complete blood count, chemistry pane, liver function tests)
- Pregnancy Test
- Concomitant medication review
- Plasma, serum, RNA, buccal, and fecal sample collection
- Adverse Event Review and Evaluation
- Randomization
- Administration of study drug
- Distribution of drug diary

Study participants will undergo follow-up study visits every 4 months. Study procedures are summarized in Table 2. Follow-up COVID-19 Questionnaire is included as Additional File 2.

**Table 2:** Study procedures

	Genotype screening visit 0^a^	Enrollment visit 1^b^	Visit 2	Visit 3	Visit 4	Visit 5	Visit 6	Visit 7/end-of-trial	Monthly phone calls	Follow-up/end-of-trial phone call	Early end of study visit
Month			4	8	12	16	20	24		EOT + 6 wks	
Targeted Day		1	120	240	360	480	600	720			
Visit Window (days)		− 28 to 1^c^	± 7	± 7	± 7	± 7	± 7	± 7	± 4	± 4	
Informed consent	X	X^d^									
Demographics	X	X^d^									
Medical history		X									
Eligibility Review	X	X									
Randomization		X									
Complete Physical exam		X									
Brief Physical exam^e^			X	X	X	X	X	X			X
Vital signs		X	X	X	X	X	X	X			X
Study Site Spirometry^f^		X	X	X	X	X	X	X			X
Home spirometry tutorial and spirometer provided		X^g^									
DLCO^f^		X	X	X	X	X	X	X			X
QOL questionnaires^h^		X			X			X			X
COVID-19 Questionnaire		X	X	X	X	X	X	X			X
Home Spirometry Survey^i^				X		X		X^j^			X^j^
Complete blood count (CBC)		X						X			X
Chemistries and liver function tests		X	X	X	X	X	X	X			X
Pregnancy Test^k^		X		X		X		X			X
Blood for genotype	X^l^										
Biomarkers and gene expression (plasma, serum, RNA, cells, buccal, fecal)		X	X	X	X	X	X	X			X
Hospitalization Assessment			X	X	X	X	X	X	X	X	X
AE review and evaluation		X	X	X	X	X	X	X	X	X^m^	X
Conmed review		X	X	X	X	X	X	X	X	X	X
Administer study drug		X	X	X	X	X	X				
Distribute Drug Diary		X	X	X	X	X	X				
Collect and Review Drug Diary			X	X	X	X	X	X			X
Study drug accountability			X	X	X	X	X	X			X

*EOT* End of treatment

Genotype Screen Participants: Participants recruited for the study without a prior confirmed TT TOLLIP genotype

Genotype Confirmed Participants: Participants that have a confirmed TT TOLLIP genotype prior to recruitment for PRECISIONS, through participation in the Pulmonary Fibrosis Foundation Registry and Biorepository or other Biorepository. Genotype has been confirmed by the Clinical Coordinating Center (CCC) and communicated to the site prior to the site contacting the participant

^a^Visit 0 (Genotype Screening Visit) will be performed for genotype screen participants only. Visit 1 may be performed only after the site reviews the genotype results to confirm eligibility. Genotype results are expected to be available to report to sites within 14 days

^b^All assessments at Visit 1 must be performed before administration of the study drug

^c^Visit 1 procedures may be completed over a 4 week time period. If a participant is found ineligible at Visit 1, but later meets criteria, they may return to the study site to complete any outstanding Visit 1 procedures within 4 weeks. If a participant does not complete all Visit 1 procedures or does not meet the eligibility criteria within 4 weeks of Visit 1, they must be scheduled for a re-screen visit. Please refer to Sect. 8.1.1 for further information

^d^Informed consent and Demographics do not have to be repeated at Visit 1 if they were completed at Visit 0 (Genotype Screening Visit)

^e^A brief physical exam will be conducted at visits 2 through 7. Brief physical exams will include assessment of general appearance, respiratory, cardiovascular, dermatologic, and abdominal system

^f^PFTs for routine clinical care done within 28 days of Visit 1 at the study site’s PFT lab may be used for baseline, only if the participant has been clinically stable over that time period. If the participant has not been stable, PFTs should be done prior to randomization in order to assess eligibility criteria

^g^Participants will be provided with a home spirometer and training. Participants will perform home spirometry during Visit 1. This procedure should not be performed if the participant opted out of home spirometry in the informed consent

^h^Leicester cough questionnaire, EuroQOL EQ-5D, UCSD-Shortness of Breath Questionnaire, K-BILD, SGRQ, R-Scale-PF

^i^Home Spirometry Survey will only be given to participants who opted in to home spirometry

^j^The Home Spirometry Exit Survey should be give at Visit 7/Early End of Study Visit

^k^Pregnancy urine dipstick testing will be done for women of childbearing potential only

^l^Genotype screen participants will have their blood drawn for genotype testing at Visit 0, only after signing the PRECISIONS study consent. Genotype results are expected to be available to report to sites within 14 days

^m^Study staff will record all new reportable events with start dates occurring any time after informed consent is obtained until Visit 7 (for non-serious AEs) or 30 days (for SAEs) after the last study visit, End of Treatment Visit (EOT) or Visit 7

### Home spirometry

Home spirometry has been shown to be feasible and practical in tracking IPF progression for patients [34]. PRECISIONS participants will be asked to perform spirometry at home three times a week (Monday, Wednesday and Friday) in the morning using hand-held, vertically mounted turbine spirometers (GoSpiro®, Monitored Therapeutics Inc, Dublin, OH, USA). Participants and study investigators will be blinded to the results of home spirometry for the duration of the study. Participants will receive feedback on quality of the maneuver by the GoSpiro® avatar. Home spirometry data will undergo quality control and quality assurances by the University of California Los Angeles (UCLA) Exercise Physiology Research Laboratory, under the supervision of Dr. Christopher Cooper, in coordination with the data coordinating center. Participation in the home spirometry study will be optional. Participants will be asked reasons for choosing not to participate in home spirometry, and will be surveyed about their experience with home spirometry during and at the end of the study (Additional files 3 and 4).

### Primary endpoints and endpoint adjudication

The primary endpoint of the PRECISIONS trial will be the time to a composite of 10% relative FVC decline, first respiratory hospitalization, lung transplantation, or all-cause mortality. Each of the events comprising the primary composite endpoint are clinically meaningful. FVC decline and hospitalizations have been linked to disease progression and subsequent time to death [35, 36]. Lung transplantation is a surrogate marker of disease progression and severity. Respiratory hospitalizations will be determined by a blinded clinical events adjudication committee.

### Secondary endpoints

Secondary endpoints include the following:

- Time to 10% relative decline in FVC% predicted, first respiratory hospitalization, lung transplantation, or death from any cause
- Time to death from any cause
- Time to first respiratory hospitalization
- Time to 10% relative decline in FVC
- Time to lung transplantation
- Time to 10% relative decline in FVC% predicted
- Time to first all-cause hospitalization
- Annualized rate of respiratory hospitalizations
- Annualized rate of non-elective, all-cause hospitalizations
- Proportion of participants undergoing lung transplantation during follow-up
- Change in FVC % predicted from randomization to 12 and 24 months
- Change in FVC from randomization to 12 and 24 months
- Change in DLCO uncorrected, from randomization to 12 and 24 months
- Change in patient-reported outcomes scores from randomization to 12 and 24 months (Leicester Cough Questionnaire, EuroQoL EQ-5D, UCSD-SOB, K-BILD, SGRQ scores)
- Proportion of participants with and number of treatment- emergent adverse events, serious adverse events, adverse events leading to discontinuation, unanticipated problems

### Exploratory endpoints

Exploratory endpoints are related to home spirometry and include the following:

- Time to 10% relative decline in home spirometry FVC
- Time to 10% relative decline in home spirometry FVC % predicted
- Change in home spirometry FVC % predicted from randomization to 12 and 24 months
- Change in home spirometry FVC from randomization to 12 and 24 months
- Comparison between a 10% relative decline captured by home spirometry with the relative change in FVC captured by the next on-site FVC measurement

### Data and safety monitoring board

Safety and oversight will be under the direction of a Data and Safety Monitoring Board (DSMB) that is appointed by the NHLBI. The DSMB will be composed of members with the appropriate expertise for this study. Members of the DSMB will be independent from the study conduct and without conflict of interest. The DSMB will meet at least semiannually to assess the safety and efficacy data for each study arm. They will review the study protocol, monitor all aspects of the study (recruitment, adverse events, protocol adherence, data quality, attrition, demographic and baseline characteristics), and recommend protocol modifications, including early study termination. Any proposed changes to the study protocol will also be reviewed by the DSMB.

### Statistical analysis plan

Continuous variables will be summarized using descriptive statistics including, n, means, standard deviations, medians, 25th and 75th percentiles, and minimum and maximum. Categorical variables will be presented as counts and percentages. Demographic and baseline summaries will be provided by treatment group and overall. Two-sided hypotheses will be tested for endpoints at the 5% level. SAS software (SAS Institute, Inc., Cary, NC, USA) and R freeware will be used to perform the analyses.

### Analysis of the primary endpoint

The primary analysis plan will be based on a modified intent-to-treat design applied to participants who have received at least one dose of study medication. A two-sample log-rank test will be used to assess whether assignment to a 24-month course of NAC is superior to placebo for the primary composite endpoint (time to 10% relative decline in FVC, first respiratory hospitalization, lung transplantation, or death from any cause). Participants will be censored at 2 years of follow-up or at loss to follow-up, whichever occurs first.

Secondary analyses include assessing treatment differences in the composite endpoint graphically using Kaplan–Meier estimates. The effect of NAC versus placebo will be explored in subgroups of interest with results tabulated (estimated hazard ratio by subgroup, corresponding 95% confidence interval and p-value) as well as displayed via forest plots. Pre-specified subgroups include concomitant therapy usage, sex, race/ethnicity, smoking history, dichotomized age, dichotomized baseline FVC (L and % predicted), dichotomized baseline DLCO (ml/min/mm Hg and % predicted) and any respiratory hospitalizations in the previous year. Multivariable analyses of the primary endpoint will be conducted based on whether proportionality of hazards holds. Cox proportional hazards models will be used if the hazards are proportional, otherwise restricted mean regression will be used. Variables adjusted for in the multivariable model will include concomitant therapy (pirfenidone or nintedanib vs. none), age, sex, baseline FVC, and baseline DLCO. These variables are potential confounders and are commonly included in multivariable analyses of IPF studies. In addition, we will adjust for home spirometry participation if the participation of home spirometry is below 90% of randomized study participants. The adjusted hazards ratio, 95% confidence interval, and p-value will be used to assess the impact of NAC versus placebo on the primary composite endpoint from the multivariable model.

If there is more than 10% loss to-follow-up, sensitivity analyses will be performed based on multiple imputation of the primary outcome. If more than 10% of participants having missing adjustment variables for the secondary analyses of the primary endpoint, then sensitivity analyses multiply imputing the missing predictors will be performed. In the event more than 10% of participants switch concomitant therapy use after randomization (ex. participant who was not using pirfenidone at start of trial starts using pirfenidone mid-way through the trial), a sensitivity analyses will be performed that uses inverse weighting methodology.

### Analysis of secondary and exploratory endpoints

The analyses for time-to-event secondary and exploratory endpoints will be similar to those outlined for the primary endpoint. Rate endpoint analyses will be based on univariable and multivariable zero-inflated negative binomial regression models. Analyses of trajectory endpoints will use a repeated measure approach that uses mixed models and incorporates all available assessments from randomization through 24 months. In order to examine the sensitivity and specificity of home spirometry values in predicting subsequent primary and secondary time-to-event outcomes, an exploratory receiver operating characteristic analysis will be performed.

### Sample size and power calculation

It is anticipated that the event rate in the placebo arm will be highly dependent on the proportion of patients enrolled at different Gender, Age, and Physiology (GAP) scores [37]. The GAP index score will likely be heavily weighted towards 3 given the availability of pirfenidone and nintedanib. Assuming an exponential distribution for the composite endpoint comparable to that from the PANTHER study, we conservatively estimate a placebo yearly event rate to be 24% for the primary composite endpoint in participants not taking concomitant therapy [11]. We have also taken into account concomitant IPF therapy and conservatively estimate that 65% of the study population will be taking one of the two anti-fibrotic medications approved for treatment. We estimate the placebo event rate among participants taking concomitant IPF medications will be lower at a 12% yearly even rate. The estimate of the magnitude of the treatment effect is based on the PANTHER pharmaco-genetic study of the *TOLLIP* TT genotype, in which the primary endpoint was also a composite endpoint (decline of at least 10% in FVC, lung transplantation, hospitalization, and/or time to death) [13]. The hazard ratio was 0.14 in the PANTHER cohort and 0.23 in the validation cohort, both of which are large effect estimates. Given the retrospective and observational nature of this study, we took a very conservative approach in estimating the treatment effect. The estimate effect size for PRECISIONS is a 66.7% event reduction between NAC and placebo. Based on these estimates and a 10% dropout rate, we plan to enroll 200 randomized participants, which will provide 90.1% power assuming a two-sided type-I error rate of 0.05. Table 3 summarizes the power estimates for various event rates and treatment effects.

**Table 3:** Statistical power assuming various event rates and treatment effects

Placebo event rate (%)	NAC event rate* (%)	Event rate reduction (%)	Power** (%)
24	6.0	75	97.5
30	7.5	75	99.3
36	9.0	75	99.8
24	8.0	66.7	90.1
30	10.0	66.7	96.0
36	12.0	66.7	98.7
24	12.0	50	62.3
30	15.0	50	73.1
36	18.0	50	80.6

*12-month event rates for the 35% of patients not taking concomitant IPF medication. The remaining 65% are assumed to have half the shown event rate in each arm based on taking IPF concomitant medication

**Power estimates assume censorship/dropout rate of 10% for the NAC vs placebo comparison in 200 randomized participants. Calculations assume a 2-sided Type-I error rate of 0.05. Follow-up is planned to be 24 months for all patients. Power calculations were based on a log-rank test with assumed event rates following an exponential distribution

An interim sample size adjustment is also incorporated into the statistical analysis plan. Upon enrollment of at least 25 placebo participants with at least 12 months of follow-up, an interim sample size adjustment analysis will be performed. If the observed one-year event rate in this group of placebo participants is below 16%, the sample size will be increased by 40 participants to maintain at least 80% power for the study assuming a 66.7% event rate reduction in the NAC arm compared with placebo. No type I error would be spent as part of this analysis because the adjustment would not take into account the event rate in the NAC arm. This approach is a precaution that protects study power in the case of the placebo arm event rate being below the range anticipated in the design of the trial.

### Additional objectives and ancillary studies

In addition to the clinical trial, a major objective of PRECISIONS is to apply blood-based ‘omics technologies and provide novel insights in the pathogenesis of pulmonary fibrosis and different types of interstitial lung disease (ILD). In collaboration with the PFF and its biorepository, PRECISIONS will perform whole genome sequencing, proteomic analyses, transcriptional profiling and other ‘omics-powered analyses on blood samples from approximately 2000 ILD patients. Accomplishing this objective may enable identification of subgroups of patients who are more responsive to specific therapies based on the underlying pathobiology, and lead to future precision medicine trials not only for IPF but also other types of ILD. This is an opportunity for investigators to leverage the data generated from PRECISIONS to catalyze new diagnostic and therapeutic developments for pulmonary fibrosis. Ancillary study and publications committees have been created to ensure a structured process in adjudicating study proposals from researchers requesting data (including biospecimens) that ensures plausibility and appropriate resource allocation.

## Discussion

### Personalizing medicine for patients with IPF

Precision medicine approaches have been adopted in the treatment of malignancy and cardiovascular disease, with an increasing number of therapies prescribed based on an individual’s clinical risk and genetic profile [38]. This approach has been lacking in pulmonary fibrosis. Patients with IPF have highly heterogenous clinical trajectories, and prognosis for each individual patient is difficult to predict with currently available clinical and molecular tools. Multiple genetic and environmental factors have been linked to susceptibility and progression of IPF [1]. These key differences among patients with IPF suggest that subgroups of patients may respond differently to treatments. The gap between basic/translational research and its application to the clinical care of IPF patients has narrowed, driven by collaborative efforts among researchers to advance the understanding of mechanisms that drive progressive lung fibrosis, and disprove ineffective treatments. PRECISIONS is rooted in a plausible mechanism that links dysregulated innate immunity to pulmonary fibrosis based on pre-clinical models, identification of genes critical in regulating immunity (i.e., *TOLLIP*), and pharmaco-genetic studies that suggest a potential benefit of NAC in IPF patients with the *TOLLIP* TT genotype. This study demonstrates the feasibility of designing an IPF clinical trial that uses a precision medicine approach and may ultimately identify an effective therapy for a subgroup of patients with this disease.

### Leveraging the PFF care center network for clinical trials and research

Since the *TOLLIP* TT genotype is only present in about 25% of patients with IPF, reaching our enrollment target of 200 participants requires screening of about 800 patients. We expect that 75% of patients with IPF will not meet the genotype requirement. To mitigate this high screen failure rate, we are leveraging existing blood samples from subjects enrolled in the PFF Patient Registry and Biorepository. The PFF Patient Registry and Biorepository prospectively enrolls participants who have been diagnosed with IPF and other interstitial lung diseases at more than 70 Care Center Network sites across the United States. The purpose of the PFF Registry and Biorepository is to increase opportunities for clinical investigation in pulmonary fibrosis by collecting clinical data and biological samples of consented patients with pulmonary fibrosis [39]. Patient recruitment for PRECISIONS will occur at approximately 25 sites selected from PFF Care Center Network, based on broad geographic coverage and participation in the Biorepository. It is hoped that PRECISIONS can provide a paradigm for how future studies can leverage the infrastructure of the PFF Care Center Network and the PFF Patient Registry to execute targeted trials in a broad and diverse population of patients with pulmonary fibrosis.

### Utility of home spirometry in future IPF trials

Endpoints in IPF clinical trials have traditionally used FVC obtained at in-person study site visits. Home spirometry has been shown to be feasible and practical in tracking disease severity and progression in patients with IPF [34, 40]. There has been significant interest in using FVC measured by home spirometry as a clinical endpoint in IPF trials. This approach may potentially allow for more frequent measurements, more precise assessments of changes in FVC, and reduced sample sizes required for clinical trials. However, the utility of home spirometry in IPF clinical trials remains uncertain [41, 42]. The home spirometry substudy of PRECISIONS provides an opportunity to investigate the most effective protocols and appropriate analysis plans that may shape future clinical trials to use home spirometry as a primary endpoint. By making the substudy optional, we will also investigate the characteristics of those patients who opt into the study, and examine barriers to home spirometry in this patient population. Data obtained from home spirometry will provide valuable information on potentially incorporating this procedure in future clinical trials.

### Future studies

The rationale and design of the PRECISIONS trial was largely shaped by data from the IPFnet PANTHER trial and other cohorts of IPF patients. The usage of stored biospecimens from clinical trials is critical in expanding our knowledge of IPF, identifying novel treatments, and determining which subgroups of IPF patients may benefit by investigative therapies. Therefore, biospecimens will be collected and stored from participants in the PRECISIONS trial for future research purposes. Blood will be collected at each study visit and will include plasma, serum, RNA, and cellular samples. Buccal and fecal samples will also be collected at each study visit. This extensive and serial collection of biospecimens will facilitate further research in IPF.

### Support

PRECISIONS is primarily funded by the National Lung Heart and Blood Institute (grant number UH3HL145266 and U24HL145265), National Institutes of Health. Additional funding sources include the Three Lakes Foundation, which is a philanthropic organization with the mission of advancing care for IPF patients by supporting efforts to improve time to diagnosis and accelerate new treatments (https://threelakesfoundation.org/). The Pulmonary Fibrosis Foundation is an integral collaborator for this clinical trial as PRECISIONS has the potential to demonstrate the utility of partnering with prospective cohorts to ensure clinical trial enrichment (https://pulmonaryfibrosis.org/).

In summary, PRECISIONS takes a novel approach to IPF clinical trials by targeting a subgroup of patients with IPF and a specific genotype that may benefit from NAC therapy based on prior pharmaco-genetic studies. In addition, the collection of various biological specimens, exploratory utility of home spirometry, and comprehensive clinical event data will provide substantial resources for future investigations of IPF and other types of ILD.

## Supplementary Information

**Additional file 1:** Baseline COVID-19 Questionnaire. COVID-19 questionnaire to be completed by all study participants at the baseline visit. PRECISIONS logo created by the PRECISIONS study team for the PRECISIONS study. Written permission obtained from the Data Coordinating Center.

**Additional file 2:** COVID-19 Questionnaire Follow-Up. COVID-19 questionnaire to be completed by all study participants at each follow-up visit. PRECISIONS logo created by the PRECISIONS study team for the PRECISIONS study. Written permission obtained from the Data Coordinating Center.

**Additional file 3:** Home Spirometry Survey. Home spirometry survey to be completed at visit 3 and visit 5 by study participants who agreed to enroll in the home spirometry substudy. PRECISIONS logo created by the PRECISIONS study team for the PRECISIONS study. Written permission obtained from the Data Coordinating Center.

**Additional file 4:** Home Spirometry Exit Survey. Home spirometry survey to be completed at visit 7 or early termination visit by study participants who agree to enroll in the home spirometry substudy. PRECISIONS logo created by the PRECISIONS study team for the PRECISIONS study. Written permission obtained from the Data Coordinating Center.

## Data Availability

Not applicable.

## Abbreviations

CCC: Clinical Coordinating Center
DLCO: Diffusing capacity for carbon monoxide
DSMB: Data and Safety Monitoring Board
FDA: Food and Drug Administration
FVC: Forced vital capacity
GAP: Gender, age, and physiology
ILD: Interstitial lung disease
IPF: Idiopathic pulmonary fibrosis
K-BILD: King’s brief interstitial lung disease
NAC: *N*-Acetylcysteine
NHLBI: National Heart Lung and Blood Institute
PAMP: Pathogen-associated molecular pattern
PANTHER: Prednisone azathioprine and NAC therapy in IPF
PFF: Pulmonary Fibrosis Foundation
PRECISIONS: Prospective Treatment Efficacy in IPF Using Genotype for NAC Selection
SGRQ: St. George’s Respiratory Questionnaire
TLR: Toll-like receptor
UCLA: University of California Los Angeles
UCSD-SOB: University of California San Diego Shortness of Breath
