# Supplementary material for: Design and rationale for the prospective treatment efficacy in IPF using genotype for NAC selection (PRECISIONS) clinical trial
Source: BMC Pulm Med. 2022 Dec 13;22:475. doi: 10.1186/s12890-022-02281-8 (PMC9746571; doi:10.1186/s12890-022-02281-8)
Supplement: Supplementary file 1 — Additional file 1: Baseline COVID-19 Questionnaire. COVID-19 questionnaire to be completed by all study participants at the baseline visit. PRECISIONS logo created by the PRECISIONS study team for the PRECISIONS study. Written permission obtained from the Data Coordinating Center. [file 12890_2022_2281_MOESM1_ESM.pdf]

PRECISIONS
Prospective tReatment EffiCacy in IPF uSIng genOtype for Nac Selection (PRECISIONS) trial

BASELINE COVID-19 QUESTIONNAIRE

Visit 1

Participant ID#: ___ ___ P ___ ___ ___ ___ ___
Date: _____ /_____ / ________

Please answer each of the questions to the best of your ability. There are no right or wrong answers.

1. Since January 2020, have you had an illness that you thought might be COVID-19?

☐ Yes ➔ When did you have an illness you thought might be COVID-19? ____/_______(mm/yyyy)

☐ No

2. Have you been told by a doctor or other healthcare professional that you had COVID-19?

☐ Yes ➔ When were you told by a doctor or other healthcare professional that you had COVID-19? ____/_______(mm/yyyy)

☐ No

3. Have you ever been tested for COVID-19?

☐ Yes ➔ **Please complete Question 3a** ☐ No ➔ **Skip to Question 4**

3a. Have you ever had a test that was positive for COVID-19?

☐ **Yes ➔ Please complete table below** ☐ **No ➔ Skip to Question 3b**

Please complete the table below with information for any <u>positive</u> COVID-19 tests you've had.

**Date of Test** (dd/mm/yyyy)	**Type of Test (check one)**
1 ___/___/_____	☐ Nasal swab ☐ Mouth swab ☐ Saliva/Spit test ☐ Blood ☐ Other, specify: ________________
2 ___/___/_____	☐ Nasal swab ☐ Mouth swab ☐ Saliva/Spit test ☐ Blood ☐ Other, specify: ________________
3 ___/___/_____	☐ Nasal swab ☐ Mouth swab ☐ Saliva/Spit test ☐ Blood ☐ Other, specify: ________________
4 ___/___/_____	☐ Nasal swab ☐ Mouth swab ☐ Saliva/Spit test ☐ Blood ☐ Other, specify: ________________
5 ___/___/_____	☐ Nasal swab ☐ Mouth swab ☐ Saliva/Spit test ☐ Blood ☐ Other, specify: ________________

 Initials of participant completing this form: _______

BASELINE COVID-19 QUESTIONNAIRE

Visit 1

Participant ID#: ___ ___ P ___ ___ ___ ___ ___
Date: _____ /_____ / ________

3b. Have you ever taken a test that was <u>negative</u> for COVID-19?

☐ Yes ➔ **Please complete table below** ☐ No ➔ **Skip to Question 3c**

Please complete the table below with information for any <u>negative</u> COVID-19 tests you've had.

**Date of Test** (dd/mm/yyyy)	**Type of Test (check one)**
1 ___/___/_____	☐ Nasal swab ☐ Mouth swab ☐ Saliva/Spit test ☐ Blood ☐ Other, specify: _______________
2 ___/___/_____	☐ Nasal swab ☐ Mouth swab ☐ Saliva/Spit test ☐ Blood ☐ Other, specify: _______________
3 ___/___/_____	☐ Nasal swab ☐ Mouth swab ☐ Saliva/Spit test ☐ Blood ☐ Other, specify: _______________
4 ___/___/_____	☐ Nasal swab ☐ Mouth swab ☐ Saliva/Spit test ☐ Blood ☐ Other, specify: _______________
5 ___/___/_____	☐ Nasal swab ☐ Mouth swab ☐ Saliva/Spit test ☐ Blood ☐ Other, specify: _______________

3c. If you were tested for COVID-19, were you tested because: (check all that apply)

- ☐ You had symptoms
- ☐ You had contact with person(s) with COVID-19
- ☐ Screening for your job in your community
- ☐ Other, Specify: ____________________________________

4. If you have had COVID-19 or thought you had COVID-19, did you have any of the following symptoms related to your illness? <u>If you have not had, or don't think you've had COVID-19, please skip to Question 9</u>.

☐ Yes ➔ **(Check all that apply)**

- ☐ Fever or chills
- ☐ Increased or new shortness of breath
- ☐ Increased or new cough
- ☐ Chest pain
- ☐ Abdominal pain
- ☐ Nausea or vomiting
- ☐ Diarrhea
- ☐ Muscle aches or joint pain
- ☐ Increased or new fatigue
- ☐ Sore throat
- ☐ Congestion or runny nose
- ☐ Headache
- ☐ Loss of smell or taste
- ☐ Confusion
- ☐ Trouble sleeping
- ☐ Conjunctivitis
- ☐ Skin changes
- ☐ Other, Specify:_____________

☐ No

Initials of participant completing this form: _______

BASELINE COVID-19 QUESTIONNAIRE

Visit 1

Participant ID#: ___ ___ P ___ ___ ___ ___ ___
Date: _____ /_____ / ________

5. Are you recovered from your COVID-19 illness now?

☐ Yes, completely ➔ How long did it take to return to your usual state of health?
# of ___________ days **OR**
# of ___________ weeks **OR**
# of ___________ months

☐ No, better but still have some problems

☐ No, still have major problems or are disabled from COVID-19

5a. <u>If you answered NO to Question 5,</u> are you still having any of the below symptoms/problems related to your COVID-19? (check all that apply)

☐ Yes ➔ **(Check all that apply)**

- ☐ Fever or chills
- ☐ Increased or new shortness of breath
- ☐ Increased or new cough
- ☐ Chest pain
- ☐ Abdominal pain
- ☐ Nausea or vomiting
- ☐ Diarrhea
- ☐ Muscle aches or joint pain
- ☐ Increased or new fatigue
- ☐ Sore throat
- ☐ Congestion or runny nose
- ☐ Headache
- ☐ Loss of smell or taste
- ☐ Confusion
- ☐ Trouble sleeping
- ☐ Conjunctivitis
- ☐ Skin changes
- ☐ Other, Specify:_____________

☐ No

5b. Are you experiencing any of the following new problems since your acute COVID-19 illness? (check all that apply)

- ☐ Problems with your memory
- ☐ Problems with paying attention
- ☐ Problems with your appetite
- ☐ Problems with feeling lightheaded
- ☐ Trouble sleeping
- ☐ Periods of racing heart
- ☐ Inability to exercise at pre-COVID level
- ☐ Inability to return to work (if you were working pre-COVID)
- ☐ Inability to return to your usual pre-COVID activities
- ☐ Feeling weak, tired, and/or sick 24-48 hours after physical activity
- ☐ Other, Specify:_____________

6. Have you had an overnight stay in a hospital due to any illness related to COVID-19?

☐ Yes ➔ How many nights did you stay in the hospital? ____________ nights

☐ No ➔ **Skip to Question 7**

 Initials of participant completing this form: _______

BASELINE COVID-19 QUESTIONNAIRE

Visit 1

Participant ID#: ___ ___ P ___ ___ ___ ___ ___
Date: _____ /_____ / ________

6a. While in the hospital, did you have any of the following?

Oxygen (by mask or nose)	☐ Yes	☐ No
A breathing tube or ventilator to help you breathe	☐ Yes	☐ No
Intensive care unit (ICU) or ICU monitoring	☐ Yes	☐ No
Dialysis	☐ Yes	☐ No

7. Were you prescribed medication(s) for COVID-19?

☐ Yes ➔ Which medication(s)? ________________________________
☐ No
☐ Don't know

8. Has a healthcare provider ever told you that you may have gotten COVID-19 a second time, or that you have been "re-infected" with COVID-19?

☐ Yes ➔ **Continue on to Questions 8a – 8d**
☐ No ➔ **Skip to Question 9**

8a. Not counting your original infection, how many more times do you think you have been re-infected with COVID-19?

☐ 1 ☐ 2 ☐ 3 ☐ 4 ☐ 5 or more

8b. When do you know or think you were first <u>re-infected</u> with COVID-19?

____/_______(mm/yyyy) *please estimate even if you are not sure

8c. At that time, what made you think you had been re-infected? (check all that apply)

☐ You had another test that showed you had COVID-19
☐ You had symptoms of COVID-19 (fever, cough, trouble breathing)
☐ You had contact with person(s) with COVID-19
☐ Other, Specify: ________________________________

8d. This time, when you were re-infected, how did your symptoms compare to your first infection with COVID-19?

☐ Worse than the first infection
☐ About the same as the first infection
☐ Better than the first infection
☐ You had no symptoms

Initials of participant completing this form: _______

PRECISIONS
Prospective tReatment EffiCacy in IPF uSIng genOtype for Nac Selection (PRECISIONS) trial

BASELINE COVID-19 QUESTIONNAIRE

Visit 1

Participant ID#: ___ ___ P ___ ___ ___ ___ ___
Date: _____ /_____ / ________

9. Have you received a vaccine for COVID-19?

☐ Yes ➔ When did you receive your first vaccine? ____/_______(mm/yyyy)
☐ No ➔ **Skip to Question 10**

9a. Did you receive a second vaccine for COVID-19?

☐ Yes ➔ When did you receive your second vaccine? ____/_______(mm/yyyy)
☐ No

9b. Are you (or were you) part of a COVID-19 vaccine research study?

☐ Yes ☐ No

9c. Which vaccine did you receive?

☐ Moderna ☐ Pfizer ☐ AstraZeneca ☐ Johnson & Johnson
☐ Unknown ☐ Other, Specify: ________________

10. This is a list of potential actions we want to know if you have taken, <u>over the past 4 months</u>, to reduce your risk of exposure to COVID-19. You can say "most or all of the time," "sometimes," or "rarely or never."

a. Staying at home	☐ Most/all times	☐ Sometimes	☐ Rarely/Never
b. Avoiding contact with people outside of my home	☐ Most/all times	☐ Sometimes	☐ Rarely/Never
c. Washing hands or using sanitizer frequently	☐ Most/all times	☐ Sometimes	☐ Rarely/Never
d. Staying at least 6 feet away from others	☐ Most/all times	☐ Sometimes	☐ Rarely/Never
e. Avoiding large gatherings	☐ Most/all times	☐ Sometimes	☐ Rarely/Never
f. Avoiding eating indoors at restaurants/bars	☐ Most/all times	☐ Sometimes	☐ Rarely/Never
g. Cancelled planned travel	☐ Most/all times	☐ Sometimes	☐ Rarely/Never
h. Wearing a face mask	☐ Most/all times	☐ Sometimes	☐ Rarely/Never
i. Not shaking hands or touching people	☐ Most/all times	☐ Sometimes	☐ Rarely/Never
j. Not going to work (working remotely)	☐ Most/all times	☐ Sometimes	☐ Rarely/Never
k. Wiping down surfaces with disinfectant	☐ Most/all times	☐ Sometimes	☐ Rarely/Never

 Initials of participant completing this form: _______
