# Supplementary material for: Design and rationale for the prospective treatment efficacy in IPF using genotype for NAC selection (PRECISIONS) clinical trial
Source: BMC Pulm Med. 2022 Dec 13;22:475. doi: 10.1186/s12890-022-02281-8 (PMC9746571; doi:10.1186/s12890-022-02281-8)
Supplement: Supplementary file 4 — Additional file 4: Home Spirometry Exit Survey. Home spirometry survey to be completed at visit 7 or early termination visit by study participants who agree to enroll in the home spirometry substudy. PRECISIONS logo created by the PRECISIONS study team for the PRECISIONS study. Written permission obtained from the Data Coordinating Center. [file 12890_2022_2281_MOESM4_ESM.pdf]

Visit 7 or Early Termination Visit

Participant ID#: ___ ___ P ___ ___ ___ ___ ___
Date: _____ /_____ / ________

Home Spirometry Exit Survey

Instructions: The following questions ask you about your experience with the home spirometry (lung function testing) during the entire course of the research study. Please make a check mark or x in the box next to your chosen answer. There are no right or wrong answers. All of this information will help us to better understand the positives as well as the challenges associated with performing the lung function testing at home in order to improve the process in the future.

1. How satisfied were you with the process of using home spirometry in this study?
   - ☐ Very satisfied
   - ☐ Satisfied
   - ☐ Can take it or leave it
   - ☐ Dissatisfied
   - ☐ Very dissatisfied

2. How did the home spirometry process in this study meet your expectations of remote monitoring of your idiopathic pulmonary fibrosis?
   - ☐ Poorly
   - ☐ Unremarkable
   - ☐ Meets expectations
   - ☐ Better than expected
   - ☐ Outstanding

3. If you answered “poorly” or “unremarkable” to Question 2, can you please provide the reasons why you feel your expectations were not met?

4. Did you face any challenges with using the home spirometer during this research study?
   - ☐ Yes
   - ☐ No

 Initials of the participant completing this form: _________

Participant ID#: ___ ___ P ___ ___ ___ ___ ___
Date: _____ /_____ / ________

Home Spirometry Exit Survey

5. If you answered YES to Question 4, please describe the challenges you have faced below:

6. How much technical difficulty did you have using the spirometry device?
   - ☐ No difficulty
   - ☐ Very little difficulty
   - ☐ Some difficulty
   - ☐ A lot of difficulty

7. What did you think of the level of feedback you received from the device about your technique?
   - ☐ Very satisfied
   - ☐ Satisfied
   - ☐ Can take it or leave it
   - ☐ Dissatisfied
   - ☐ Very dissatisfied

8. Did a family member or caregiver help you with the test?
   - ☐ Yes
   - ☐ No

9. How motivated were you to use the home spirometry during this research study?
   - ☐ I was very motivated
   - ☐ I was somewhat motivated
   - ☐ I was not motivated at all

10. Were you been able to keep up with using the home spirometry three days per week as directed?
    - ☐ Yes
    - ☐ No

Initials of the participant completing this form: _________

Participant ID#: ___ ___ P ___ ___ ___ ___ ___
Date: _____ /_____ / ________

Home Spirometry Exit Survey

11. Did you think performing home spirometry 3 days per week during this research study was:
    - ☐ Too little
    - ☐ Too much
    - ☐ Just the right amount

12. If you did not choose "just the right amount", how often would you suggest performing home spirometry during this research study and why?

13. How likely are you to recommend this mode of testing to other patients with idiopathic pulmonary fibrosis?
    - ☐ Not likely
    - ☐ Somewhat likely
    - ☐ Very likely

14. Which do you prefer, using the home spirometer or having the test performed in clinic?
    - ☐ Home
    - ☐ Clinic
    - ☐ Equal, no preference

15. What suggestions do you have to improve your experience using the home spirometry during this research study?

16. Would you want to continue the use of home spirometry after the completion of this study?
    - ☐ Yes
    - ☐ No

Initials of the participant completing this form: _________
